# Dystonia during pegylated interferon alpha therapy in a case with essential thrombocythemia and cerebral infarction

**DOI:** 10.1007/s10072-024-07829-6

**Published:** 2024-10-23

**Authors:** Peng Zhang, Bin Han, Kun Meng, Xiao Cheng, Yi Liu, Yaxuan Sun, Fengyun Hu

**Affiliations:** Neurology Department, Shanxi Provincial People’s Hospital, The Fifth Hospital of Shanxi Medical University, 29, Double Towers Temple Street, Taiyuan, Shanxi Province China

Dear editor,

Pegylated interferon has been reported to cause dystonia, possibly involving alterations in the basal ganglia circuitry and dopamine pathways. 1 Dystonia was reported to be prominent in only 2 reported cases (Table 1) [1, 2].

**Table 1:** Clinical features of our patient and previously reported cases of pegylated interferon-associated dystonia

Reference	Age/gender	Original disease	Dose of pegylated Interferon	Time of symptom onset	Clinical manifestation	Flu-like illness	Treatment and curative effect	Prognosis
Patient	42y/M	Essential Thrombocyt-hemia(ET)	Total dose 135 µg /week	2–3 months after the first dose of interferon	Right arm was raised, even flexion to the shoulder and back, while left forearm was up and left hand was stretched out, and it was difficult to move lower limbs	Fever	Clonazepam,1 mg, per os, the symptoms completely resolved within 20 min	There was no recurrence of dystonia
1	24y/M	Chronic hepatitis B and delta infections	1.5 µg/kg weekly; total dose 120 µg /week	2 h after the first dose of interferon	Involuntary movements of the neck and extremities, predominantly of the arms, and lingual protrusion that lasted up to 20 min	Mild to moderate signs of flu-like illness	Diazepam,5 mg, intramuscular injection, the symptoms completely resolved within 30–40 min	Dystonia did not reappear at this time
2	31y/M	Chronic hepatitis C virus infection	1.5 µg/kg weekly; total dose 105 µg /week	6 h after the first dose of interferon	Pisa syndrome (pleurothotonus) with retrotorticollis, associated with rest and action trembling	None	Biperiden,2 mg, intramuscular injection, the symptoms completely resolved approximately 30 min	The patient did not develop any similar reaction subsequently

We present a 42-year-old male, he suddenly developed numbness of his right hand in April 2023, with the right limbs weakness, and gradually lost some of the verbal expression ability. He was taken to a local hospital, where the patient was treated with alteplase 64.8 mg intravenous thrombolysis immediately after cranial computed tomography(CT) showed no bleeding. There was a transient improvement in the right limbs weakness after the treatment, but the symptoms worsened again. Brain magnetic resonance imaging (MRI) examination revealed multiple acute infarctions in the left cerebral hemisphere and the M1 segment occlusion of the left middle cerebral artery (Fig. 1A, B). The doctor had considered mechanical thrombectomy, but the blood cell analysis report showed thrombocytosis (666 × 10^9/L). After learning that he had 10 years of thrombocytosis without regular treatment, the endovascular treatment of cerebral artery occlusion was excluded by the doctor because of the high risk of reocclusion after mechanical thrombectomy. Based on thrombocytosis, the patient underwent myelopuncture and tested for MPN related gene mutations and fusion detection. Results were shown as positive for the JAK 2 mutant gene, NM_004972:c. 1849G> (p.V617F)exon14, Variation frequency of 23.2% (1771X). He was therefore diagnosed with Essential Thrombocythemia, treated with a total dose of polyethylene glycol-2b interferon 135µg every 10 days, and developed flu-like symptoms such as fever.

**Fig. 1:**
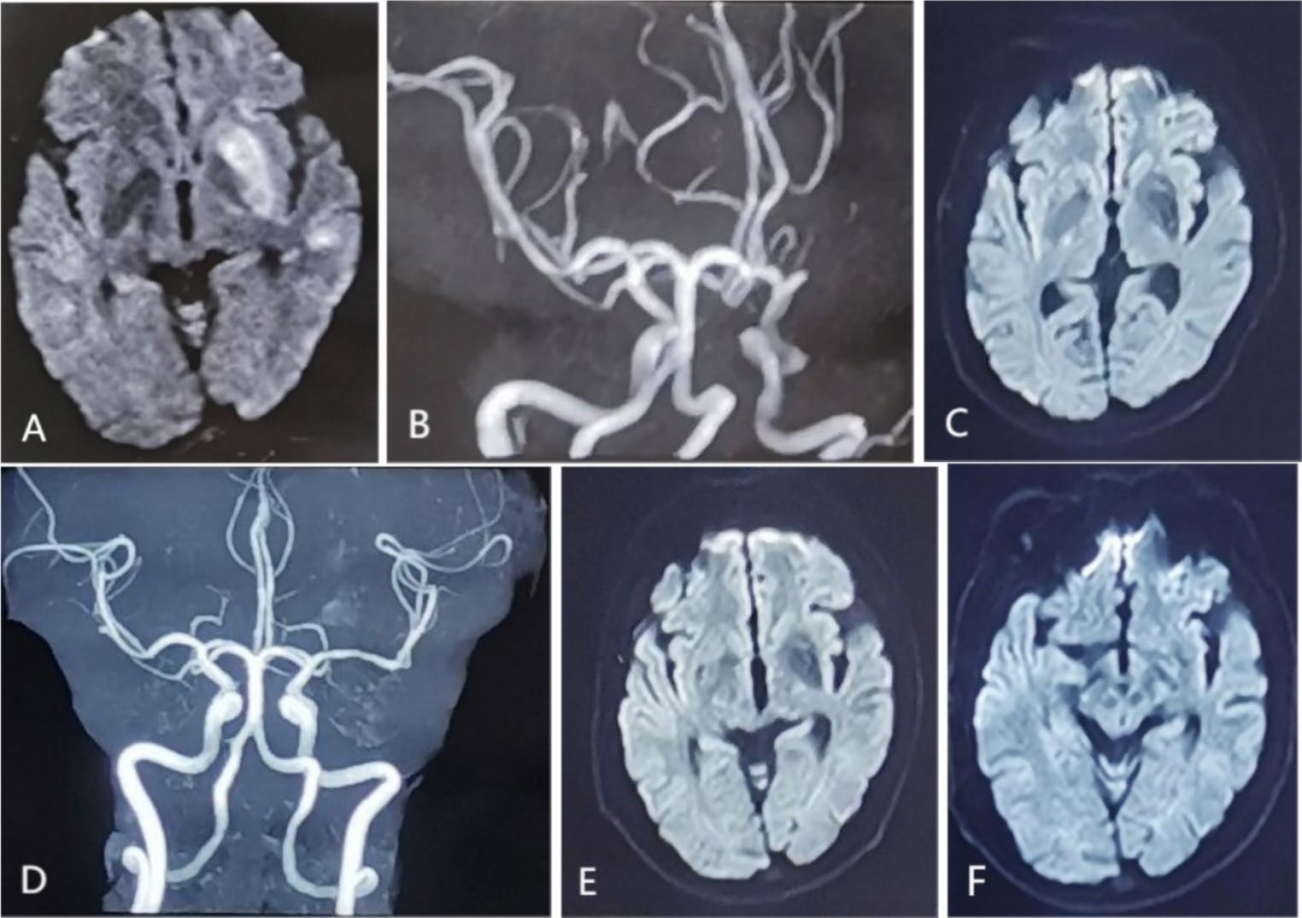
Magnetic resonance during the course of the disease. **A**: DWI showed left basal ganglia and temporal lobe hypersignal shadow (April 3, 2023); **B**: MRA displayed the M1 segment occlusion of the left middle cerebral artery(April 3, 2023); **C**: DWI revealed no abnormal high signal(July 21, 2023); **D**: MRA showed recanalization of the left middle cerebral artery occlusion segment; **E**: DWI revealed no abnormal high signal(December 5, 2023); **F**: DWI revealed no abnormal high signal(April 11, 2024)

After discharge, the patient regularly took oral aspirin enteric-coated tablets 100 mg/daily and atorvastatin calcium tablets 20 mg/daily. In July of the same year, he was able to walk independently, with poor upper lift strength and right upper hand grip strength, but he developed transient dystonia, which was elevated in the left forearm and left hand back extension, which lasted for about 10 s. Repeat diffusion weighted imaging(DWI) revealed no abnormal high signal (Fig. 1C), and magnetic resonance angiography(MRA) showed recanalization of the left middle cerebral artery occlusion segment (Fig. 1D). In October, he changed to receive the treatment once a month because of excessive interferon costs. In December, his symptoms became worse. When moving from quiet to active, he showed abnormal movements of his limbs, showing a stiff and fixed posture(video 1-paragraph 1). *His right arm was raised and even flexion to the shoulder and back*,* while his left forearm was up and left hand was stretched out*,* and his legs legs were so stiff that they were difficult to move*,* this posture could disappear after quiet and rest; sensory trick and tremor were not evident.* The process lasted about 2 min and occurred 2–3 times. A repeat DWI displayed no abnormal high signal(Fig. 1E). He experienced severe anxiety disorder and required swearing to discharge his urine.

In April 2024, as long as he turned to activity, dystonia will develop. Similarly, DWI showed no abnormal high signal (Fig. 1F). Blood cell analysis, liver function test, kidney function test, coagulation function test, thyroid function test, human immunodeficiency virus(HIV) and syphilis antibody were normal, hepatitis B surface antibody and core antibody were all positive. No abnormality was observed on the electroencephalogram(EEG). High resolution magnetic resonance imaging vascular wall imaging presented mild stenosis of the bilateral middle cerebral arteries. *Next-generation sequencing of dystonia gene panel was performed*,* and it did not disclose any related mutations.* The symptoms completely resolved after administration of clonazepam, 1 mg per os within 20 min(video 1-paragraph 2). For subsequent treatment, clonazepam was adjusted to 0.5 mg per dose, twice a day, and no more symptoms occurred. Our patient with a negative family history, had no prior psychiatric history of illness and he had not been treated with antipsychotics. He also strongly denied the use of antipsychotic drugs.

Our case again confirms the strong association of interferon and dystonia. The difference from the two previously reported cases is characterized by the later occurrence of dystonia, about 2–3 months later; Moreover,

the symptoms of dystonia continue. If clonazepam is temporarily stopped once, dystonia will appear again, but the degree is reduced. One point to be explained is that the onset of dystonia and cerebral infarction were more than 3 months apart, and we consider no direct correlation; Critically, the degree and frequency of dystonia development increase with the extension of the interferon use cycle. Furthermore, this case shows that primary thrombocytosis is an uncommon cause of ischemic stroke. The recanalization of the occluded cerebral artery after active interferon treatment is a meaningful finding. We can infer that the presence of hepatitis B antibodies in the patient indicates that he had been infected with the virus, and perhaps interferon also plays a role in the inhibition of the virus.

## Electronic supplementary material

Below is the link to the electronic supplementary material.

Supplementary Material 1

Supplementary Material 2

Supplementary Material 3

Supplementary Material 4

Supplementary Material 5

Supplementary Material 6

## Data Availability

The authors confirm that the data supporting the findings of this study are available within the article [and/or its supplementary materials].
