# Supplementary material for: Dystonia during pegylated interferon alpha therapy in a case with essential thrombocythemia and cerebral infarction
Source: Neurol Sci. 2024 Oct 23;45(12):5943–5. doi: 10.1007/s10072-024-07829-6 (PMC11554768; doi:10.1007/s10072-024-07829-6)
Supplement: Supplementary file 1 — Supplementary Material 1 [file 10072_2024_7829_MOESM1_ESM.docx]

**VIDEO**

interferon-associated dystonia. Segment 1: Before the treatment, the video shows the patient showing involuntary limb movements and abnormal posture, his right arm up, while the left forearm and the left hand back extension, both lower limbs step difficult. Segment 2: Patient on clonazepam therapy, 1 mg per os, with complete remission of gait dystonia; The right limb movement disorder is a symptom of cerebral infarction.
