# Supplementary material for: Dystonia during pegylated interferon alpha therapy in a case with essential thrombocythemia and cerebral infarction
Source: Neurol Sci. 2024 Oct 23;45(12):5943–5. doi: 10.1007/s10072-024-07829-6 (PMC11554768; doi:10.1007/s10072-024-07829-6)
Supplement: Supplementary file 3 — Supplementary Material 3 [file 10072_2024_7829_MOESM3_ESM.docx]

Dear Editors:

We submit our manuscript entitled “*Dystonia During Pegylated Interferon Alpha Therapy in a Case With Essential Thrombocythemia and Cerebral Infarction*” to *Journal of Neurology*.

We the undersigned declare that this manuscript entitled “Dystonia During Pegylated Interferon Alpha Therapy in a Case With Essential Thrombocythemia and Cerebral Infarction” is original, has not been published before and is not currently being considered for publication elsewhere. We confirm that the manuscript has been read and approved by all named authors and that there are no other persons who satisfied the criteria for authorship but are not listed. We further confirm that the order of authors listed in the manuscript has been approved by all of us. We understand that the Corresponding Author is the sole contact for the Editorial process. He is responsible for communicating with the other authors about progress, submissions of revisions and final approval of proofs.

In this work, we evaluated a patient with Essential Thrombocythemia who received interferon therapy after a sudden cerebral infarction and developed dystonia during treatment. This case is typical and includes primary thrombocytosis, which is a rare cause of cerebral infarction, the reopening of the occluded cerebral artery after interferon treatment, and, even more rarely, interferon-related dystonia. We hope that more people will be informed of these phenomena. Hopefully this paper is suitable for the "*Journal of Neurology*".

Thank you very much for your attention and consideration.

Sincerely yours,

Peng Zhang

Contact information:

Peng Zhang

Address: Shanxi Provincial People’s Hospital,Neurology Department, 29, Double Towers Temple Street, Taiyuan, China.

1. mail: [2545785855@qq.com](mailto:2545785855@qq.com)

Mobile: +86 18834180218

**Acknowledgments:** We thank Ms.Liu Xiaoling for her contribution. This work was supported by Shanxi Key Laboratory of Brain Disease Control. There are no financial conflicts of interest to disclose.
