# Supplementary material for: Dystonia during pegylated interferon alpha therapy in a case with essential thrombocythemia and cerebral infarction
Source: Neurol Sci. 2024 Oct 23;45(12):5943–5. doi: 10.1007/s10072-024-07829-6 (PMC11554768; doi:10.1007/s10072-024-07829-6)
Supplement: Supplementary file 5 — Supplementary Material 5 [file 10072_2024_7829_MOESM5_ESM.pdf]

金域医学
KingMed Diagnostics

分子遗传检测报告

GZ2408J3AHCQA0

分子遗传检测报告

金域医学 KingMed Diagnostics

姓名:	周云会	样本类型:	全血	住院/门诊号:	
性别:	男	样本条码:	0811985345	床号:	NA
年龄:	42岁	样本实验号:	NP26S08161	电话:	NA
送检医生:	NA	家庭样本:	NA	采样时间:	NA
送检科室:	门			接收日期:	2024年08月02日
送检医院:	山西省人民医院			报告日期:	2024年08月23日
				报告版本:	V1.6

检测项目：肌张力障碍相关基因测序检测

检测技术： 对受检者基因组DNA进行肌张力障碍相关基因（见附表）目标区域捕获和测序

分析内容： 基于二代测序数据对受检者肌张力障碍相关基因上单核苷酸变异、小片段插入缺失变异及部分大片段拷贝数变异进行分析

送检原因：

NA

临床信息：

肌张力障碍

检测结论： 未检测到可以明确解释受检者表型的致病或疑似致病变异

检测结果：

在受检者的检测数据中未发现可以解释其主要临床表现的变异。

需要注意的是，本检测仅针对目前已知与 肌张力障碍相关基因 进行检测 (详见“检测范围”部分）。其数据解读局限于当前对疾病的认识（文献资料和数据库信息等）和临床提供的病史信息。此外，本检测所用技术本身具有一定的适用范围（详见“局限性声明”部分）。因此，本次阴性检测结果不能完全排除本受检者表征系基因变异所致。

建议受检者进行遗传咨询，以完整、正确地理解报告内容。

表一：可以解释受检者表型的（致病/疑似致病）变异：

本表内变异所致疾病的临床表征及预期遗传模式分别与受检者主要临床表型及合子类型相符，且其变异评级为疑似致病或致病。建议临床医生高度关注并综合临床实际，进行疾病管理、遗传咨询、生育风险评估/控制等的工作。

- 1.1 单核苷酸变异及小片段插入缺失变异（SNV/Indel）

基因	染色体位置	变异信息	合子类型	疾病名称	遗传模式	变异来源	变异分类
无							

分子遗传检测报告

金域医学 KingMed Diagnostics

姓名:	周云会	样本类型:	全血	住院/门诊号:	
性别:	男	样本条码:	0811985345	床号:	NA
年龄:	42岁	样本实验号:	NP26S08161	电话:	NA
送检医生:	NA	家庭样本:	NA	采样时间:	NA
送检科室:	门			接收日期:	2024年08月02日
送检医院:	山西省人民医院			报告日期:	2024年08月23日
				报告版本:	V1.6

- 1.2 大片段拷贝数变异（CNV）

染色体区带	变异类型	区域内相关基因	相关疾病	变异来源	变异分类
无					

表二：与受检者主要临床表型相符，但尚无法明确与其起病或病情进展间相关性的变异：

本表内变异所涉疾病的主要临床表征与受检者主要临床表型相符，但其变异评级为临床意义未明或该疾病的预期遗传模式与检测到的合子类型不符。本表也包括致病性明确、预期遗传模式与合子类型相符，但所致疾病外显率低或临床表现个体差异较大的变异。

- 2.1 单核苷酸变异及小片段插入缺失变异（SNV/Indel）

基因	染色体位置	变异信息	合子类型	疾病名称	遗传模式	变异来源	变异分类
无							

- 2.2 大片段拷贝数变异（CNV）

染色体区带	变异类型	区域内相关基因	相关疾病	变异来源	变异分类
无					

表三：仅与受检者部分临床表型相关的其他变异：

下表所列发现，与受检者 **部分临床表型相关** ，目前无法完全解释该发现与受检者表型的关联，供临床医生参考，包含以下情况：1）所涉疾病的预期遗传模式与检测到的合子类型不符，或在家系中没有呈现完全基因型-表型共分离；2）所涉疾病的主要临床表征与受检者主要临床表现不甚相符。

基因	染色体位置	变异信息	合子类型	疾病名称	遗传模式	变异来源	变异分类
PDE10A	chr6: 166076188	NM_001385079.1:c.112G>C(p.Ala38Pro)	杂合	常染色体显性遗传性纹状体变性2型 [MIM:616922]	AD	NA	临床意义未明

分子遗传检测报告

金域医学 KingMed Diagnostics

姓名:	周云会	样本类型:	全血	住院/门诊号:	
性别:	男	样本条码:	0811985345	床号:	NA
年龄:	42岁	样本实验号:	NP26S08161	电话:	NA
送检医生:	NA	家庭样本:	NA	采样时间:	NA
送检科室:	门			接收日期:	2024年08月02日
送检医院:	山西省人民医院			报告日期:	2024年08月23日
				报告版本:	V1.6

主检实验室：广州金域医学检验中心有限公司　联系电话：4001-111-120　地址：广州国际生物岛螺旋三路10号

签字:

报告主检 陈梦帆　　报告审核 黄滢玲　　报告批准 [illegible]

报告声明：

本报告对高通量数据的分析依赖于受检者及其临床医师提供的表型及病史信息、相关数据库信息和现有文献资料。本检测结果只对本次检测所接受的样本负责，仅报告与送检目的及疾病表型相关的变异结果。本检测为临床实验室自建项目（LDT），不属于国家药品监督管理局的医疗器械注册范围。如对本次检测结果有疑问，请与金域医学客服（4001-111-120）联系反馈。

局限性声明：

1. 本项目应用序列捕获高通量测序技术，对人类基因组上肌张力障碍相关基因编码区域进行测序。所获的二代测序数据在本检测所含核基因的外显子及其上下游5bp序列上的平均测序深度为大于等于90X，测序深度大于20X的序列比例为约98%。本方法不能完全覆盖重复区域及富含GC区域。此外，本方法适用于单核苷酸变异、小片段插入缺失变异及大片段拷贝数缺失和重复变异，但不适用于基因动态变异、复杂重组以及未影响基因拷贝数的杂合性缺失（Absence of Heterozygosity，AOH）变异等特殊类型变异的检测，也不适用于检测基因组结构变异、存在高度同源性序列（包括假基因）的基因区域中的变异、大片段插入变异及位于基因调节区及内含子区±5bp以外的变异。

2. 本检测所用技术方法在内部验证中对大于100kb的拷贝数变异检测敏感度为99%，对小于100kb的拷贝数变异检出率取决于基因组结构和相关区域的测序质量。本次检测中使用该方法分析所得拷贝数变异的准确性及范围均未经独立的拷贝数分析实验进行确认。因此，本结果仅供临床医生参考，需经针对拷贝数检测的实验验证方可用于诊断及临床决策的依据。

3. 本检测中不会报告所有识别的变异，仅报告已知致病相关基因中有足够证据表明能够或可能引起送检表型相关疾病的变异。对于报告中非明确致病变异，需结合临床综合判断，不宜直接作为临床决策依据。对于良性或疑似良性的变异不会报告。对于隐性遗传疾病，没有 SNV/Indel 发现的前提下，不报告 CNV 的携带者状况。

4. 本检测应用的 DNA 源自受检者血液细胞，而非源自体细胞或生殖细胞，因此不能排除体细胞嵌合现象所致的解读偏差。若血液细胞无法获得，检测的 DNA 源自受检者的组织细胞时，不能排除体细胞嵌合现象所致的检测或解读偏差。本检测不适用于存在污染可能的样本，默认所获的样本均来自相应受检者。

5. 本检测默认受检者与其受检亲属间具备生物学意义上确切相应的亲属关系，且报告不涉及血亲关系。

6. 本检测结果仅报告与申请时的临床症状相关的变异。

7. DNA 序列分析的目的是了解疾病病因或评估遗传风险。鉴于目前人类对疾病认识水平的局限性，本检测结果不排除受检者表型可由多基因变异所致。对检出的特定基因变异，在某些情况下，可能并非受检者的唯一致病因素，完整的解释有待新的研究与发现。若本检测未检出能够解释受检者表型的特定基因及致病变异位点，即检测结果为阴性。但阴性的检测结果并不能排除受检者存在某种疾病的可能性，仍然存在其它未知基因或难以检测到、或无法确定的基因变异类型或非遗传因素参与其中。

8. 本检测技术及相关仪器并非常规临床检测项目，目前主要用于辅助临床诊断等相关目的。此外，同其它检验方法一样，基因检测亦存在由于技术、样本以及操作所致低概率的假阴性或假阳性的风险。本检测报告不可用于临床最终诊断。其结果需经临床医师结合各方面情况进行综合判断。

检测方法：

- **测序实验：** 本检测在 Illumina 测序平台上完成，由金域医学建立并进行验证。
- **二级分析：** 本检测主要采用 GATK 软件套装进行测序数据分析。测序片段通过 BWA 与 UCSC hg19 参考基因组进行比对。
- **单核苷酸变异和小片段插入缺失变异分析：** 本检测采用 VEP 软件（Variant Effect Predictor）对变异进行注释，同时基于 ClinVar，OMIM，HGMD 和 gnomAD 等遗传性疾病数据库、变异数据库及人群大规模测序数据库对变异进行筛选，同时采用多种公认计算机算法对变异可能致病性进行预测和分类。需注意的是，变异注释过程使用的各数据库可能定期更新，因此在分析本例数据时，存在最新的文献证据未同步到相关数据库中的可能。
- **检测结果命名和解读：** 序列变异依据 HGVS 制定的规范进行命名。报告中单核苷酸变异和小片段插入缺失变异参考美国医学遗传学与基因组学学会（ACMG）和美国分子病理协会（AMP）发布的《序列变异解读标准和指南》进行分类。
- **拷贝数变异分析：** 采用内部专业软件基于高通量测序数据进行拷贝数变异分析。报告中拷贝数变异评级参考 ACMG 和 ClinGen 联合发布的《组成型拷贝数变异解读与报告技术标准和指南》进行分类。

检测范围

- **检测基因列表**

ACOX1	*ACTB*	*ADAR*	*ADCY5*	*AFG2B*	*AFG3L2*
ALS2	*AMPD2*	*ANO3*	*APTX*	*ARSA*	*ARX*
ATM	*ATP13A2*	*ATP1A2*	*ATP1A3*	*ATP6AP2*	*ATP7B*
B4GALNT1	*BCAP31*	*C19orf12*	*CACNA1A*	*CACNA1B*	*CACNA1G*
CBS	*CHCHD2*	*CHMP2B*	*CLN3*	*CLN5*	*CLN6*
CLN8	*COASY*	*COQ8A*	*COX15*	*COX20*	*CP*
CSTB	*CYP27A1*	*DCAF17*	*DCTN1*	*DDC*	*DDX3X*
DLAT	*DLD*	*DNAJC12*	*DNAJC6*	*EARS2*	*ECHS1*
FA2H	*FBXO7*	*FITM2*	*FOXG1*	*FTL*	*FUCA1*
FUS	*GALC*	*GCDH*	*GCH1*	*GLB1*	*GM2A*
GNAL	*GNAO1*	*GRIK2*	*GRIN2B*	*HACE1*	*HEXA*
HIBCH	*HPCA*	*HPRT1*	*HTRA2*	*IFIH1*	*KCNA2*
KCNMA1	*KCNQ2*	*KCTD17*	*KIF1A*	*KIF1C*	*KMT2B*
L2HGDH	*LRPPRC*	*LRRK2*	*MARS2*	*MAT1A*	*MCOLN1*
MECP2	*MECR*	*MMADHC*	*MMUT*	*MPV17*	*MRE11*
NDUFA12	*NDUFAF6*	*NDUFS1*	*NDUFS4*	*NDUFS7*	*NDUFS8*
NKX2-1	*NKX6-2*	*NPC1*	*NPC2*	*OPA3*	*PANK2*
PARK7	*PCCA*	*PCCB*	*PCDH12*	*PDE10A*	*PDE2A*
PDGFB	*PDGFRB*	*PDHA1*	*PDHX*	*PINK1*	*PLA2G6*
PLP1	*PNKD*	*PNKP*	*PNPT1*	*POLG*	*POLR3A*

POLR3B	*PRKN*	*PRKRA*	*PRRT2*	*PSEN1*	*PTS*
QDPR	*RAB39B*	*RNASEH2A*	*RNASEH2B*	*RNASEH2C*	*RNASET2*
SAMHD1	*SCP2*	*SDHAF1*	*SERAC1*	*SETX*	*SGCE*
SLC16A2	*SLC18A2*	*SLC19A3*	*SLC20A2*	*SLC2A1*	*SLC30A10*
SLC39A14	*SLC6A3*	*SLC6A8*	*SNCA*	*SPG11*	*SPR*
SUCLA2	*SUOX*	*SURF1*	*SYNJ1*	*TACO1*	*TAF1*
TH	*THAP1*	*TIMM8A*	*TOR1A*	*TPK1*	*TPP1*
TRAPPC11	*TREM2*	*TREX1*	*TUBB4A*	*VAC14*	*VAMP1*
VAMP2	*VPS13A*	*VPS13D*	*VPS35*	*WARS2*	*WDR45*
WDR73	*XK*	*XPR1*	*YY1*	*ZNF142*	

参考文献

- Zech M, Jech R, Boesch S. Monogenic variants in dystonia: an exome-wide sequencing study. Lancet Neurol, 2020, 19(11):908-918.
- Powis Z, Towne MC, Hagman KDF, et al. Clinical diagnostic exome sequencing in dystonia: Genetic testing challenges for complex conditions. Clin Genet, 2020, 97(2):305-311.
- Wu MC, Chang YY, Lan MY, et al. A Clinical and Integrated Genetic Study of Isolated and Combined Dystonia in Taiwan. J Mol Diagn, 2022, 24(3):262-273.
- Karczewski K J, Francioli L C, Tiao G, et al. The mutational constraint spectrum quantified from variation in 141,456 humans[J]. Nature, 2020, 581(7809): 434-443.
- Richards S, Aziz N, Bale S, et al. Standards and guidelines for the interpretation of sequence variants: a joint consensus recommendation of the American College of Medical Genetics and Genomics and the Association for Molecular Pathology[J]. Genetics in medicine, 2015, 17(5): 405-423.
- Riggs E R, Andersen E F, Cherry A M, et al. Technical standards for the interpretation and reporting of constitutional copy-number variants: a joint consensus recommendation of the American College of Medical Genetics and Genomics (ACMG) and the Clinical Genome Resource (ClinGen) [J]. Genetics in Medicine, 2020, 22(2): 245-257.
- Stenson P D, Mort M, Ball E V, et al. The Human Gene Mutation Database (HGMD®): optimizing its use in a clinical diagnostic or research setting[J]. Human genetics, 2020, 139(10): 1197-1207.

金域医学
KingMed Diagnostics

广州金域医学检验中心有限公司
广州市国际生物岛螺旋三路10号 4001-111-120 www.kingmed.com.cn
