# Supplementary material for: Dystonia during pegylated interferon alpha therapy in a case with essential thrombocythemia and cerebral infarction
Source: Neurol Sci. 2024 Oct 23;45(12):5943–5. doi: 10.1007/s10072-024-07829-6 (PMC11554768; doi:10.1007/s10072-024-07829-6)
Supplement: Supplementary file 6 — Supplementary Material 6 [file 10072_2024_7829_MOESM6_ESM.docx]

1. We revisited and modified the description of dystonia and added some details.
2. We performed genetic testing on the patients.
